# The Impact of the Availability of ECG on Treatment Time in the North Norfolk Older People Service, Norfolk and Suffolk NHS Foundation Trust

**DOI:** 10.1192/bjo.2025.10523

**Published:** 2025-06-20

**Authors:** Onyeka Nwankwo, Oluwaseun Olaluwoye, Roseline Abiola Samuel, JolaaJesu Famurewa, Abosede Ighomereho

**Affiliations:** Norfolk and Suffolk NHS Foundation Trust, Norwich, United Kingdom

## Abstract

**Aims:** The electrocardiogram is a non-invasive test used to assess cardiac function. Certain psychotropic and antidementia medications can cause bradycardia, heart block, or prolonged cardiac repolarization, worsening pre-existing conditions. Evaluating cardiac function before treatment initiation is essential. However, in the North Older People Service (NOPS), the lack of electrocardiogram availability at the time of assessment has led to significant delays in treatment initiation, particularly for patients referred for cognitive assessments. These delays not only affect individual patients but also reduce clinic efficiency, limiting access for other patients awaiting assessment.

Aims were to evaluate how the availability of ECG at the time of patient’s assessment impact on the commencement of treatment.

**Methods:** A retrospective review of electronic health records was conducted for patients referred to the North Older People Service, between January and February 2023. Of 62 accepted referrals, 5 patients were deceased, and 2 had not yet been assessed, leaving 55 for analysis. Data collected included referral dates, assessment dates, electrocardiogram availability, whether an electrocardiogram was required before treatment, treatment initiation dates, and diagnoses.

**Results:** Of the 55 patients analysed, 70.9% were started on new medications, while 29.1% were not due to mild cognitive impairment, existing treatments, or diagnoses such as vascular dementia. Among those who commenced treatment:

10.25% had an electrocardiogram at assessment and were started on treatment immediately.

71.79% did not require an electrocardiogram and were initiated on treatment without delay.

17.94% required an electrocardiogram before treatment initiation. Of these, 85.7% had dementia.

The waiting period ranged from 4 weeks and 6 days to 30 weeks and 2 days, with an average delay of 18 weeks and 2 days.

**Conclusion:** The findings support the hypothesis that the absence of an electrocardiogram at the time of assessment contributes to significant treatment delays, particularly for dementia patients. To address this issue, referring clinicians should include electrocardiograms in pre-assessment investigations, and the triaging team should ensure that electrocardiograms are requested when necessary. Implementing these measures could reduce delays, improve efficiency, and enhance patient outcomes.

